# Basophils control T cell priming through soluble mediators rather than antigen presentation

**DOI:** 10.3389/fimmu.2022.1032379

**Published:** 2023-02-09

**Authors:** Christian Möbs, Martin Salheiser, Fabian Bleise, Marie Witt, Johannes U. Mayer

**Affiliations:** Department of Dermatology and Allergology, Philipps-Universität Marburg, Marburg, Germany

**Keywords:** basophil, dendritic cell, allergy, Type 2 immunity, antigen presentation

## Abstract

Basophils play an important role in the development of type 2 immunity and have been linked to protective immunity against parasites but also inflammatory responses in allergic diseases. While typically classified as degranulating effector cells, different modes of cellular activation have been identified, which together with the observation that different populations of basophils exist in the context of disease suggest a multifunctional role. In this review we aim to highlight the role of basophils play in antigen presentation of type 2 immunity and focus on the contribution basophils play in the context of antigen presentation and T cell priming. We will discuss evidence suggesting that basophils perform a direct role in antigen presentation and relate it to findings that indicate cellular cooperation with professional antigen-presenting cells, such as dendritic cells. We will also highlight tissue-specific differences in basophil phenotypes that might lead to distinct roles in cellular cooperation and how these distinct interactions might influence immunological and clinical outcomes of disease. This review thus aims to consolidate the seemingly conflicting literature on the involvement of basophils in antigen presentation and tries to find a resolution to the discussion whether basophils influence antigen presentation through direct or indirect mechanisms.

## Introduction

**Figure 1:**
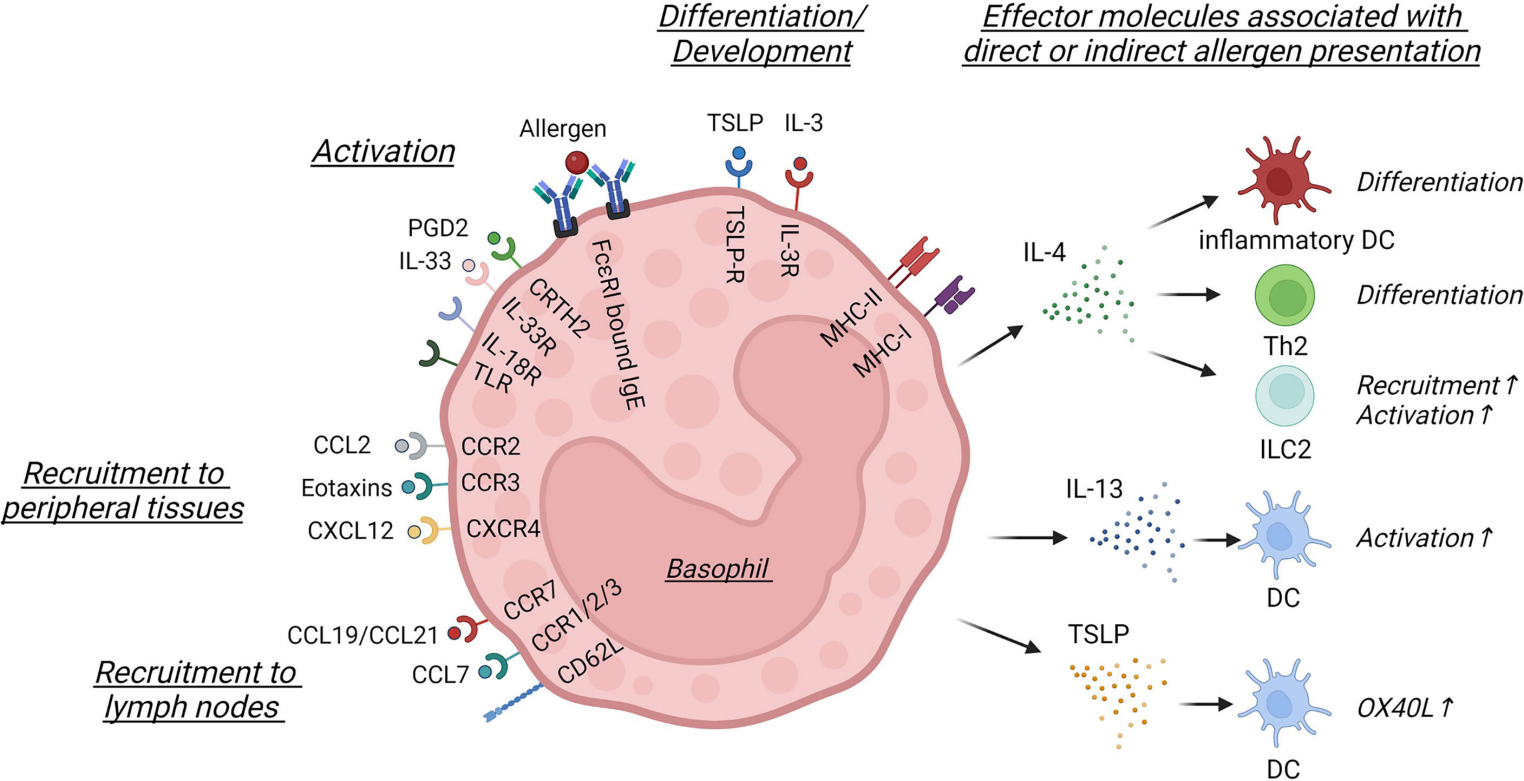
Basophil activation and effector signals involved in direct or indirect allergen presentation. In this schematic only surface and secreted molecules discussed within this review are shown. Basophil differentiation and development is controlled by TSLP and IL-3, which leads to the differential expression of cytokine and chemokine receptors, such as CRTH2, IL-33R, IL-18R, different TLRs, CCR2, CCR3 or CXCR4. Basophils can be activated by crosslinking of FcϵRI-bound IgE or by different soluble mediators, such as PGD2 or IL-33. Basophils are recruited into peripheral tissues *via* CCL2, eotaxins or CXCL12, while CCL7 signaling or CCR7 and CD62L expression facilitate lymph node entry. While in specific contexts basophils can express MHC-I and MHC-II, they are best known for the secretion of soluble mediators. IL-4 can influence the differentiation of inflammatory dendritic cells (DC) and Th2 cells or activate innate lymphoid cell type 2 (ILC2), while IL-13 and TSLP secretion activates DC and induces OX40L upregulation, indirectly influencing the priming of Th2 cells. This figure was created using biorender.

Basophils were discovered by Paul Ehrlich in 1879 during staining experiments with peripheral blood and represent the least common granulocyte population in mammals, accounting for 0.5-1% of circulating leukocytes. They differentiate from hematopoietic progenitor cells (Lin^-^CD34^+^FcϵRI^high^c-kit^-^) in the bone marrow under the control of the transcription factors C/EBPα and GATA-2 and leave the bone marrow as mature circulating basophils (1). Basophils were traditionally considered to be circulating counterparts of tissue-resident mast cells based on their expression of the high-affinity IgE receptor (FcϵRI), mechanisms of degranulation and histamine release upon activation. Facilitated by the discovery of distinct developmental pathways that are controlled by the key transcription factor C/EBPα (2), the distinct expression of c-kit/CD117 on human and murine mast cells but not basophils (3), and the development of basophil-specific transgenic knockout strains (4), a specialized role for basophils in various diseases and protective immunity have become better understood. Basophils have been shown to play an important role in allergic diseases, autoimmunity, parasitic infections and tissue homeostasis through the production of key cytokines and their interaction with immune and non-immune cells both in pro-inflammatory and anti-inflammatory contexts (5). Basophils are best studied in the context of allergy, where they have been implicated in several disease mechanisms, such as delayed IgE-mediated chronic allergic inflammation (6, 7), eosinophil entry (8), itch (9), and alternative macrophage activation (10), but also wound healing (11) and microbial dysregulation (12). Basophil activation is also used in the clinical diagnosis of allergic diseases and in monitoring the therapeutic response to immunomodulatory treatments (13). Basophils can be activated via various IgE-dependent and -independent pathways leading to the release of effector molecules like histamine, amphiregulin, eicosanoids (e.g. LTC4), granzyme B and a variety of different cytokines (e.g. IL-3, IL-4, IL-5, IL-6, IL-13, IL-25, IL-31) (14) (**Figure 1**).

The ‘classical’ activation of human and murine basophils in the context of allergy is caused by the crosslinking of FcϵRI via IgE and leads to rapid degranulation and the release of preformed histamines and proteases, followed by a secondary *de novo* synthesis of lipid mediators and cytokines and their secretion (15). Alternative activation is readily achieved *in vitro* and independent of IgE crosslinking and mediated by innate stimuli including epithelial derived inflammatory cytokines, growth factors, eicosanoids, metabolites and TLR ligands (16).

Basophils can promote allergic immune responses by producing substantial amounts of pro-allergic IL-4 and IL-13 upon allergen stimulation (17, 18), thus representing an important accessory cell type to promote Th2-like responses (19, 20). Basophils can also contribute to a Th2 bias in pro-inflammatory environments, as basophil recruitment into tumor-draining lymph nodes was found to correlate with Th2 inflammation and reduced survival in pancreatic cancer patients (21). Basophils can however also contribute to pro-inflammatory immune responses through the production of IL-6, influencing Th17 immunity. In murine models of pro-inflammatory lung inflammation basophils and their production of IL-6 contributed to the differentiation of Th17 cells (22), while in models of kidney fibrosis CXCR2^+^ basophils, recruited into the inflamed kidney, were an important source of IL-6 and controlled the number of Th17 cells (23). In human patients, basophils have also been identified in Th17-associated disorders, such as kidney fibrosis (23), IBD (24) and cystic fibrosis (25), indicating that basophils influence both Th2 and Th17 immunity through the release of key cytokines.

Beyond their role as cytokine-producing cells, basophils have also been suggested to influence the priming of adaptive immune responses by acting as unconventional antigen-presenting cells. In this review we will therefore discuss if basophils can influence antigen-presentation through direct and indirect mechanisms and correlate experimental evidence obtained in murine studies with clinical observations.

### Subsets of basophils

Four populations of circulating basophils can be identified in the blood of healthy individuals based on their surface marker expression of CD16, CD244 and FceRI (26). FceRI-expressing basophils are highly responsive to IgE and IL-3 stimulation, while FceRI^low^ basophils respond poorly to those stimuli *in vitro* (26). Resting and activated human basophils also express distinct chemokine receptors, potentially supporting their migration towards sites of inflammation or the draining lymph nodes (dLN) (27). In the context of local inflammation, murine models have shown that eotaxin-CCR3, CCR2-CCL2 and CXCR4-CXCL12 interactions are the most common (28) (**Figure 1**). Chemokine receptor upregulation can be induced by different molecular mechanisms. CXCR4 upregulation is regulated by thymic stromal lymphopoietin (TSLP) and IL-3, cytokines essential for the development and activation of basophils (29, 30), and leads to basophil migration towards a CXCL12 gradient in inflamed skin (31). In *Lyn^-/-^* lupus prone mice CXCR4 surface expression is however controlled by PGD2 signaling and leads to the accumulation of basophils in secondary lymphoid organs impacting the severity of disease (32) (**Figure 1**).

Importantly, murine basophils can be differentiated into distinct basophils subsets by *in vitro* stimulation with certain cytokines, indicating that the cytokine milieu can influence basophil maturation and effector function of basophils differently. TSLP-cultured basophils showed higher expression of IL-3R, IL-33R and IL-18Rα and less degranulation, while producing higher levels of IL-4, IL-6, CCL3 and CCL12 in the context of IL-3, IL-18 and IL-33 activation (33) (**Figure 1**). IL-3-cultured basophils showed higher expression of CD11b and CD62L, higher production of chemokines and produced more TNFα, suggesting a pro-inflammatory differentiation (33). A similar heterogeneity was observed in human basophils, which developed in a TSLP-elevated environment during food allergy-associated eosinophilic esophagitis (EoE) (30). While expression levels of HLA-DR, CD28, CD40, CD86, CD69 and CD203c were similar to those observed in healthy donors, basophils from EoE patients expressed significantly higher levels of the IL-33R, indicating that different basophil populations are associated with an altered susceptibility to allergic inflammation (33). In patients with mild to moderate asthma, basophils were strongly activated by TSLP leading to secondary production of IL-3, suggesting that in certain contexts TSLP and IL-3 can also act in concert (34).

Phenotypically different subgroups of basophils have also been observed in patients with chronic urticaria when analyzing both the frequency of peripheral basophils and their reactivity to certain stimuli. Here, stimulation of peripheral blood basophils with anti-FcϵRI revealed distinct reactivity patterns. While one group of patients exhibited a concentration-dependent activation of basophils (responders), FcϵRI stimulation failed to activate basophils in the non-responder group (35, 36). This incapability to induce IgE-mediated reactions despite sufficient FcϵRI might be due to a lack of expressing the tyrosine kinase Syk and/or an overexpression of the Src-homology 2-containing-5’-inositol phosphatases (SHIP)-1 and SHIP-2, pathways which control FceRI signaling (35, 37). Among the nonreactive patients, a subgroup with pronounced basopenia (basophils accounting for less than 0.1% of peripheral blood cells) could been identified (38). The basophils of this clinically most severely affected cohort were characterized by a significantly augmented background activation, reduced receptor-bound IgE and a decrease in surface expression of FcϵRI (39). Basopenia was associated with more severe disease, whereas the basophil responder phenotype was associated with longer disease duration.

Decreased frequencies of circulating basophils are furthermore observed in other disorders, such as allergic contact dermatitis, bullous pemphigoid, systemic lupus erythematosus or atopic dermatitis (AD) (40–42), and are likely caused by their migration into the affected tissues or secondary lymphoid organs (32). This is supported by evidence that transient basopenia reflects basophil migration to the skin during skin irritation (43) or the bronchoalveolar lavage fluid upon aeroallergen challenge (44) and might be controlled by similar or distinct chemotactic pathways compared to anaphylaxis (45).

Within tissues, basophils not only drive classical symptoms of allergic inflammation *via* histamine and leukotriene release, but also impact a number of immunological mechanisms *via* cytokine production, making them a highly immunologically relevant cell type (46) (**Figure 1**).

### Direct mechanisms of basophil-enhanced antigen presentation

Whether basophils have antigen-presenting capacity is still debated and has been reviewed before (47, 48). Mice deficient in interferon-regulatory factor 2, a transcription factor believed to suppress basophil differentiation, show a marked increase in basophil numbers and develop spontaneous Th2 responses (49). Another molecule, Lyn kinase controls basophil GATA3 expression and *Lin^-/-^* mice exhibit basophilia and a basophil-dependent Th2 bias (50), indicating an important role for basophils in driving type 2 immunity. In *Lin^-/-^* mice but also in the context of parasite infection and certain allergy models, murine basophils have been reported to express MHC-II (20, 51–53), suggesting their involvement in antigen-presentation. While MHC-II expression of murine basophils could also be observed in certain hapten-induced models of type 2 immunity (53, 54), basophils examined in models of airway and skin allergy did not express MHC molecules (55, 56). Similar observations were made in allergic patients, where no expression of HLA-DR was observed in patients allergic to house dust mite (HDM), birch pollen as well as in healthy individuals before or after *in vitro* stimulation (57–60). Yet, patients from an allergen-rich environment displaying aFUT6 deficiency (effectively reducing the ability of basophils to egress from the blood stream and infiltrate tissues) developed reduced itch sensitivity and lower amounts of HDM-specific IgE, indicating that basophils influence Th2 immunity (61). While the mechanisms of antigen-presentation were not investigated further in this study, MHC-II expression by basophils might be regulated by the cytokine milieu or affect the development of distinct basophil subsets with distinct expression patterns. However, the reported MHC-II surface expression in murine basophils was several orders of magnitude lower than those observed in B cells and dendritic cells (DC) (51), highlighting that carefully controlled isolation and analysis protocols are necessary to avoid contaminated readouts (62).

**Figure 2:**
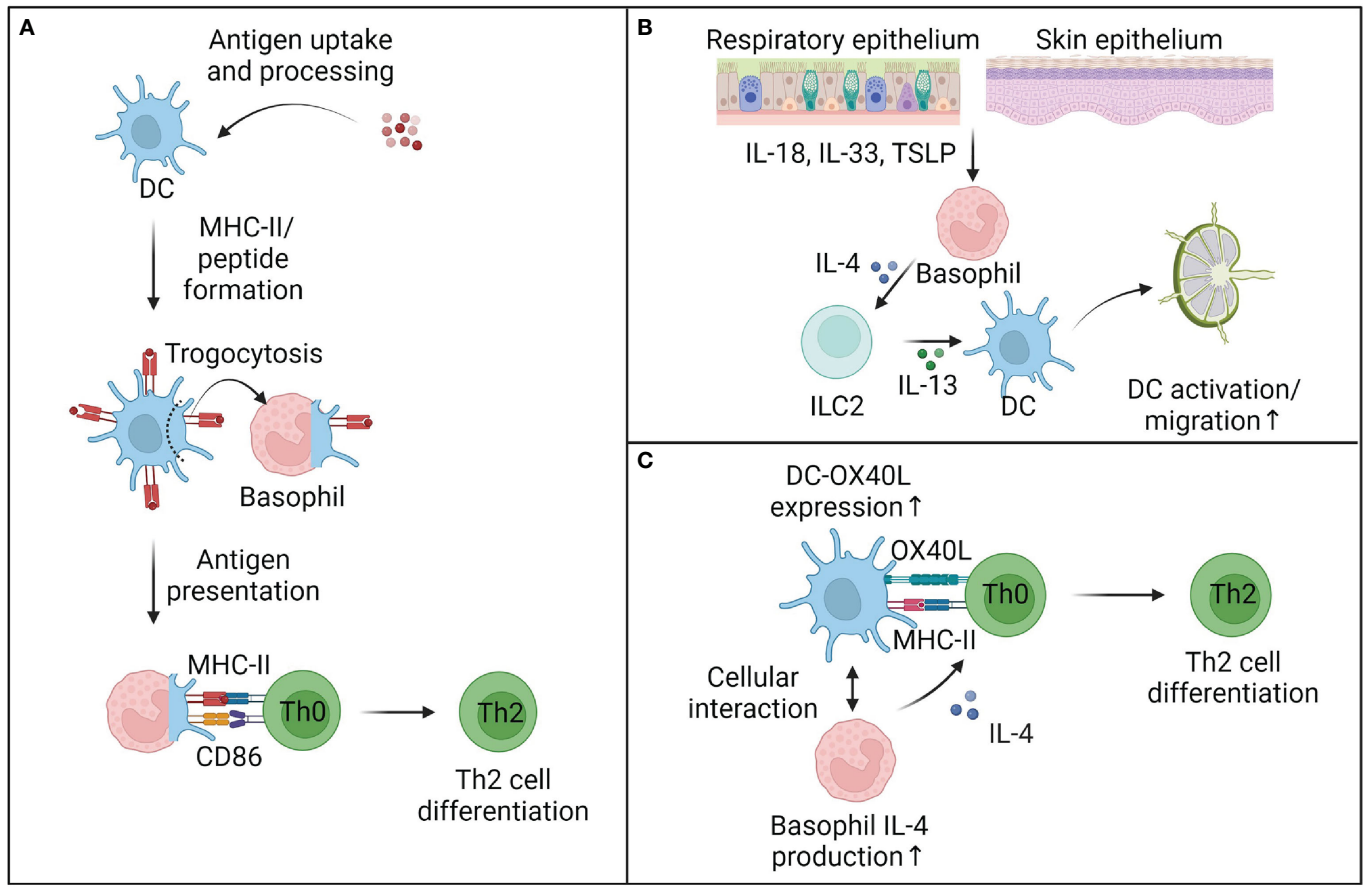
Mechanisms of basophil-enhanced antigen presentation. **(A)** Basophils can cooperate with dendritic cells (DC) to prime T cells. While basophils cannot take up and process complex antigens, they can trogocytose parts of cell membranes and antigen-loaded MHC-II complexes from DC and thus directly influence Th2 cell differentiation. It remains unclear to what extent trogocytosis plays a role *in vivo*, but other mechanisms of basophil-enhanced antigen presentation are well described. **(B)** In tissues, basophil-derived IL-4 activates murine innate lymphocytes type 2 (ILC2), which produce IL-13 and other mediators and activate DC to migrate to the draining lymph nodes. **(C)** Within lymph nodes, basophils can enhance DC activation and OX40L expression, while also providing early IL-4 to support the differentiation of Th2 cells. Although the requirement of early IL-4 for the differentiation of Th2 cells is debated, multiple studies provide evidence that basophils directly support the priming of Th2 cells, at least in the murine system. This figure was created using biorender.

While the tools to assess antigen uptake *in vivo* are limited, uptake of natural and model antigens has not been observed in murine and human basophils (29, 55, 58), while antigen-processing could be observed in certain *in vitro* settings (54, 63). Bone marrow-cultured murine basophils generated *in vitro* using IL-3 and GM-CSF showed a substantial increase of MHC-II molecules on their surface. While no corresponding increase in MHC-II transcript levels could be measured in basophils, it was observed that DC, which expressed high levels of MHC-II and were also developing under the same culture conditions, provided a possible source for MHC-II protein (47). Further experiments between purified bone marrow-derived basophils and DC confirmed that MHC-II molecules were derived from DC and acquired by basophils through cell contact-dependent trogocytosis (63) (**Figure 2A**). While the molecular requirements facilitating basophil-specific trogocytosis are not well understood, trogocytosis has been observed in other immune cells, either involving uptake of cellular membrane from dead cells, resulting in killing or active cellular membrane transfer (64). The process most similar to trogocytosis observed between basophils and DC is the interaction between T cells and DC. Here, trogocytosis requires ligand-receptor interaction between the T cell receptor (TCR) and a matching peptide-MHC complex (65). This interaction leads to the formation of an immunological synapse resulting in the internalization of the TCR and the transfer of peptide-MHC complexes, together with membrane fragments of DC onto the surface of the T cell (66, 67). This mechanism has been observed for both CD4^+^ and CD8^+^ T cells (68) and TCR-mediated trogocytosis is dependent on both actin polymerization and the TCR signaling pathway (69) and can be impaired by blocking costimulatory molecules or integrin interactions (70). While TCR-mediated trogocytosis can be excluded as a mechanism for basophils, it remains to be determined if integrin binding facilitates trogocytosis between basophils and DC.

Trogocytosis might also enhance the expression of costimulatory molecules by basophils. Naïve as well as stimulated murine basophils can express several costimulatory markers like CD40, CD80 and CD86 (20, 53, 71), but in contrast to DC do not upregulate these markers upon stimulation (54). While murine basophils constitutively express CD80 and CD86, co-culturing with DC further increases surface CD86, which might be linked to cell membrane trogocytosis (63). While trogocytosis has not been studied in the context of human basophils, basophils extracted from healthy individuals or allergic patients did not express costimulatory molecules, neither after being freshly isolated nor when stimulated with cytokines, IL-3, antigens or TLR agonists (58–60).

Several studies have shown that basophils can drive Th2 polarization *in vitro*, when purified from immunized mice and pulsed with OVA peptide (18, 20). While not being able to process full length proteins, murine basophils can present and cross-present OVA peptides efficiently and induce CD4 as well as CD8 T cell proliferation *in vitro* (53, 71), indicating that basophils have a certain capacity for antigen presentation. After depletion of basophils using an anti-FcϵRI-directed MAR-1 antibody, Th2 responses were also decreased *in vivo* in an MHC-II-dependent manner (19, 20), suggesting a direct role of basophil-mediated antigen presentation. However, Hammad et al. demonstrated that *in vivo* basophil depletion with an anti-FcϵRI MAR-1 antibody had strikingly different effects on subsequent Th2 challenge with HDM allergen compared to anti-CD200R3 (Ba103) antibody treatment, because of the depletion of FcϵRI^+^ inflammatory DC (55). While originally classified as monocyte-derived DC, these inflammatory DC have recently been identified as FcϵRI^-^ FcγRIV-expressing cDC2, which are depleted by the MAR-1 antibody due to its cross-reactivity with FcγRIV (72, 73). More specific depletion models of basophils using the anti-CD200R3 antibody or transgenic mouse models under the control of *Mcpt8* could show that basophils were not required for the development of Th2 cells in models of parasite infection (29, 56, 74) and models of airway or skin allergy (55, 75, 76), despite cellular interactions between basophils and T cells being observed (77). These studies made clear that DC were essential for T cell proliferation and Th2 priming, disproving earlier claims (54). In addition, these results also aligned with findings from patients samples, which showed that antigen-pulsed basophils purified from PBMC of healthy donors or allergic patients could not drive T cell proliferation in CFSE assays in contrast to other antigen-presenting cells (57–60). While these studies cannot exclude a cooperation between basophils and other cell types, basophils seem to have a limited capacity to drive T cell responses independently.

### Indirect mechanisms of basophil-enhanced antigen presentation

Several mechanisms have been reported, which describe how basophils cooperate with other immune cells to enhance antigen presentation. In particular, the cooperation between basophils innate lymphoid cell type 2 (ILC2) and DC has been defined as an important immune axis in type 2 immunity (**Figure 2B**). Tissue ILC2 have been shown to play a complex role in allergic inflammation of both the lung and the skin (78, 79) and are found in close proximity with basophils in skin biopsies of AD patients and in pre-clinical models of AD. It could be observed that basophils and ILC2 form clusters in inflamed skin, with basophil accumulation preceding ILC2 activation and proliferation (80). Similar to IL-4-dependent accumulation of lung ILC2 during parasite infection (81), skin ILC2 accumulation was dependent on basophil-derived IL-4 in the murine MC903-induced model of AD (80). Basophil-derived IL-4 also controls the function of ILC2 in allergic lung inflammation through the production of IL-13 and the recruitment of eosinophils (82). IL-13 has in turn been shown to be major activator of DC both in the skin and lung (78, 83, 84), suggesting an indirect cooperation between basophils and DC *via* ILC activation in the skin and lung.

Basophils have also been reported in dLN, where they are localized within the T cell zone (19, 85). Basophils recruitment to the dLN is driven by TSLP signaling, although it remains unclear if TSLP acts on DC or T cells to recruit basophils or drives the development of a dLN-migratory basophil subset (33, 76, 86, 87). Basophil entry into the dLN is facilitated by CD62L and CCL7, which support basophil binding to high endothelial venules and migration into the T cell zone (19, 75). Similarly, CD62L and CCR7 were upregulated in basophils from newly diagnosed systemic lupus erythematosus patients and associated with their accumulation in secondary lymphoid organs (42). Basophils have also been shown to enhance humoral immunity and together with CD4^+^ T cells, profoundly enhanced B cell proliferation and immunoglobulin production (88).

It has been suggested that basophils can present antigen under certain contexts, but this mechanism might be less relevant for initial Th2 cell priming, as much fewer basophils are found in the dLN compared to DC and are recruited to the dLN at later timepoints (55). These findings are supported by observations that basophils isolated from healthy human spleens showed no expression of HLA-DR or costimulatory molecules at steady state or after *in vitro* stimulation and could not drive T cell proliferation, indicating that human basophil function is restricted to the secretion of soluble mediators (89). However, other studies have suggested that basophils provide help to DC for optimal Th2 induction (75, 90, 91). As basophils are major producers of IL-4, while DC are not (92), basophils could provide an early source of IL-4 (93), especially in dLN(**Figure 2C**). IL-4 has also been suggested to activate DC and induce the differentiation of inflammatory DC (94) observed in allergic and viral inflammation (55, 73). *In vitro* co-cultures between IL-4-deficient basophils, DC and OT-II T cells showed that Th2 cell differentiation was reduced and OX40L expression by DC was decreased in the absence of basophils or basophil-derived IL-4 (95). Furthermore, Di et al. underline the importance of OX40L signaling by DC and basophils. Blocking OX40-OX40L interactions with an anti-OX40L antibody strongly reduced allergic airway inflammation following OVA sensitization and adoptive transfers of OVA-challenged basophils into OX40^-/-^ mice or blockade of OX40L led to reduced lung inflammation (96). As the requirement for an initial source of IL-4 in Th2 priming continues to be critically debated (97–100), regulation of OX40L expression through basophils might represent an additional mechanism of how basophils can influence antigen presentation (**Figures 1**, **2C**).

## Discussion

In the early 2000s an interesting hypothesis developed, which suggested that basophils could drive Th2 immunity independently of DC, and supply signals for antigen presentation, costimulation and Th2 polarizing cytokine secretion (20, 54, 101). This led to multiple studies investigating this hypothesis in different models of parasite infection, skin and lung allergy, which found that basophils could not process and present complex protein antigens, where present in dLN in much lower numbers than DC and arrived at later timepoints (55, 56, 74). Similarly, basophils collected from allergic patients, were not able to internalize, process or present allergen and thus failed to induce proliferation and cytokine secretion in T cells (57, 58). In line with this, basophils are unlikely directly involved in the priming of *de novo* Th2 cells, but could enhance DC activation and Th2 priming through the production of IL-4, the activation of ILC or other mechanisms of cellular cooperation [as reviewed in (47, 89, 90, 102)].

In patients, different populations of basophils have been observed in a range of human diseases including tumors, fibrosis, infection and chronic inflammation (5), and it is unknown if under certain conditions human basophils obtain antigen-presenting capacities, especially in the context of antigen challenge or chronic disease. Multiple murine studies have shown that basophils enhance T cell responses after antigen challenge (22, 103), yet little is known regarding human diseases, due to limited studies in affected tissues. While many studies agree that basophils do not express MHC-II or HLA-DR transcript, cell contact-dependent acqusition of MHC-II through trogocytosis could represent an additional molecular mechanism that allows basophils to be involved in antigen presentation. While trogocytosis has been studied in murine bone marrow-derived basophils (63), it is unknown if it also occurs *in vivo*, affects human basophils and also other surface molecules reported on basophils, including costimulatory molecules or MHC-I (20, 53, 71).

Additional studies to understand the molecular mechanisms that lead to the differentiation of basophil populations in the context of disease are therefore urgently necessary. While it is difficult to follow basophil differentiation during the progression of disease, seeding of basophils into tissue organoids from control- or patient-derived samples might offer new opportunities to study cellular differentiation and mechanisms of cellular cooperation and trogocytosis.

As basophils represent very rare immune cells, improved protocols to isolate basophils from affected tissues are also necessary to characterize basophils with novel technologies like single-cell sequencing. These analyses should however not only focus on transcriptomic signatures (e.g. by using single-cell RNA sequencing), but be combined with surface protein detection, such as site-seq or high-dimensional flow cytometry, to capture functional molecules that might have been acquired from other cells types. These studies might highlight tissue- and disease-dependent differences between basophil populations that contribute to disease and indicate their relationship to basophils within tissues in comparison to circulating basophil populations (104). As basophils have a multifaceted immunological role, these studies might ultimately define subpopulations that drive specific disease phenotypes through direct or indirect antigen presentation, cytokine secretion or histamine/leukotriene release, and allow for their selective targeting in the context of disease.

## Author contributions

CM, MS, FB, MW and JM were involved in the writing of the original manuscript. MS, FB, and JM were involved in creating the figures. CM and JM were responsible for revisions and editing. All authors contributed to the article and approved the submitted version.
